# Generation and Characterisation of Novel Pancreatic Adenocarcinoma Xenograft Models and Corresponding Primary Cell Lines

**DOI:** 10.1371/journal.pone.0103873

**Published:** 2014-08-22

**Authors:** Anna B. Wennerström, Inger Marie Bowitz Lothe, Vandana Sandhu, Elin H. Kure, Ola Myklebost, Else Munthe

**Affiliations:** 1 Cancer Stem Cell Innovation Centre and Department of Tumour Biology, Institute of Cancer Research, The Norwegian Radium Hospital, Oslo University Hospital, Oslo, Norway; 2 Department of Genetics, Institute of Cancer Research, The Norwegian Radium Hospital, Oslo University Hospital, Oslo, Norway; 3 Department of Pathology, Oslo University Hospital, Oslo, Norway; 4 Department for Environmental Health and Science, Telemark University College, Telemark, Norway; Garvan Institute of Medical Research, Australia

## Abstract

Pancreatic adenocarcinoma is one of the most lethal cancer types, currently lacking efficient treatment. The heterogeneous nature of these tumours are poorly represented by the classical pancreatic cell lines, which have been through strong clonal selection *in vitro*, and are often derived from metastases. Here, we describe the establishment of novel pancreatic adenocarcinoma models, xenografts and corresponding *in vitro* cell lines, from primary pancreatic tumours. The morphology, differentiation grade and gene expression pattern of the xenografts resemble the original tumours well. The cell lines were analysed for colony forming capacity, tumourigenicity and expression of known cancer cell surface markers and cancer stem-like characteristics. These primary cell models will be valuable tools for biological and preclinical studies for this devastating disease.

## Introduction

The 5-year overall survival of patients with pancreatic cancer is a dismal 6.7%. Although overall mortality for all cancers has fallen from 210 to 171 pr. 100 000 US citizens from 1992 to 2010, the overall mortality for pancreatic cancers is unchanged. So, even though pancreatic cancers account for less than 10% of the diagnosed tumours, it is the 4^th^ leading cause of cancer-related deaths in the US (www.cancer.gov). The high mortality may partly be due to the abdominal localisation where tumours may advance without early symptoms, and are diagnosed late in the disease progression, well after the acquisition of the aggressive nature of this cancer type [1]. Thus, pancreatic cancers are often metastatic and resistant towards irradiation and chemotherapy at the time of diagnosis, with a corresponding lack of efficient treatment options for the patients. The most common malignant pancreatic tumours are the pancreatic ductal adenocarcinomas (PDACs), originating from epithelial cells lining the pancreatic ducts, accounting for more than 85% of pancreatic tumours. PDACs can be of the pancreatobiliary or intestinal type, where the pancreatobiliary is the most common [2], [3]. The pancreatobiliary type tumours mostly consist of glandular and duct-like structures, well to moderately developed, growing in a desmoplastic stroma. The poorly differentiated tumours form densely packed, small irregular glands as well as solid sheets and individual cells. The intestinal type adenocarcinoma form simple or cribriform glands, and are similar to the adenocarcinomas of the large intestine in growth pattern and differentiation. The degree of differentiation in pancreatic tumours has been found to be an independent prognostic marker to the same degree as tumour size and lymph node status [4]. The “cancer stem cell” hypothesis has been under intense investigation over recent years, and in many cancer types cells with stem cell characteristics are found to generate tumours much more efficiently upon injection in mice than do bulk tumour cells [5]. These so-called “cancer stem-like cells” (CSC) have the capacity for self-renewal as well as the capacity to differentiate, and have an increased resistance to cancer treatments like chemotherapy and irradiation. Altogether, these characteristics permit CSCs to generate metastases as well as treatment-resistant recurrences. Several candidate pancreatic CSC markers have been identified, including cell surface markers CD24/CD44/CD326 [6] or CD133 [7], side population positive cells [8], and cells with aldehyde dehydrogenase activity [9].

At present, there is a lack of relevant model systems to study clinically important subpopulations of tumour cells, e.g., stem-like cells, in pancreatic cancers. Few pancreatic cancer cell lines are available, and those commonly used have been grown in culture for extended periods of time. Long term *in vitro* cultivation may induce a selective pressure to adapt to the culture conditions and the cell lines thereby no longer represent the original heterogenic tumours whereby the cells may gain mutations or altered programming *in vitro*. Furthermore, many of the classical pancreatic cell lines were generated from metastatic tumours [10]. Few primary models are available due to the limited amounts of available surgical material. In addition, there are problems generating *in vitro* cultures due to the high stromal infiltrations in pancreatic tumours, from which rapidly growing fibroblasts tend to overgrow the adenocarcinoma cells. To overcome these difficulties we generated xenografts from surplus operation material from patients with primary pancreatic tumours and thereafter established cell lines from these xenograft-passaged tumours. The original tumours and the xenografts show the same histology regarding growth pattern and differentiation. All but one tumour that generated xenografts were of the pancreatobiliary type, three moderately differentiated and three poorly differentiated. The last tumour was a moderately differentiated PDAC of intestinal type. All the generated cell lines matched the original tumours' fingerprints, had global mRNA expression pattern resembling their corresponding original tumours and were tumourigenic when injected into NOD/SCID mice. We characterised these cell lines for cell surface expression of markers known to be important for tumourigenicity and potential cancer stem cell markers during *in vitro* passaging. A schematic overview of the workflow performed in this study is found in fig. S1.

## Materials and Methods

### Ethics statements

The study was approved by the Regional Committee for Medical and Health Research Ethics South-East Norway (265-08412c) and the Institutional Review Board of Oslo University Hospital, and performed according to the guidelines of the Helsinki Convention. Informed written consent was obtained from all patients. Animal work was performed according to protocols approved by the National Animal Research Authority in compliance with the European Convention of the Protection of Vertebrates Used for Scientific Purposes (approval ID 3275 and 3530; http://www.fdu.no/). All surgery was performed under sevofluran anaesthesia, and all efforts were made to minimize suffering.

### Mutation analysis

The *KRAS* mutations (codons 12 and 13) were determined using the Wobble-enhanced amplification refractory mutation system [11].

### Pathology/Immunohistochemistry

The macro- and microscopic pathology work followed a standardised protocol. Experienced pathologists set the final diagnoses in accordance with the WHO Classification of Tumors of the Digestive System [12]. The tumours were sub-classified into pancreatobiliary and intestinal type as first described by Kimura *et al.* [13]. Furthermore, the adenocarcinomas were classified according to site of origin and tumour stage, in accordance with the pTNM Classification of Malignant Tumours [14].

The patient and xenograft tumours were subjected to semi-quantitative evaluation by immunohistochemistry (IHC). Four µm whole sections from formalin-fixed paraffin-embedded (FFPE) tissue were mounted on Menzel Super Frost Plus glass slides. For deparaffinisation and heat-induced epitope retrieval, Dako PT link was used (Dako, Glostrup, Denmark). The immunostaining was performed on the Dako EnVision+ detection system. The Dako EnVision Flex+Target Retrieval Solution at high pH was used for preheating for all the antibodies, except for Snail+Slug antibody, where low pH was used. The slides were rinsed in Dako wash buffer according to the manufacturer's instructions. Endogenous peroxidase activity was blocked for five minutes by 0.03% H_2_O_2_, washed twice in Dako wash buffer and then incubated with primary antibody at room temperature for 30 minutes before an additional wash step. The next step was 30-minute incubation with the appropriate HRP-labelled polymer conjugated secondary antibodies at room temperature before wash and ten minute incubation in diaminobenzidine (DAB). The last steps were rinsing twice in water, counterstaining with haematoxylin and mounting in Diatex. The protocol was optimised for each antigen. CK7 (Dako cat. 7081, 1∶300), E-Cadherin (Invitrogen, CA, USA cat. 13-1700, 1∶3000), Snail+Slug (AbCam, Cambridge, UK, cat ab63371, 1∶100), Vimentin (Dako cat. M0851, 1∶1600), CD326 (EpCAM) (Dako cat. M3525, 1∶60). P53 (Santa Cruz Biotechnology, SC, USA, cat sc-126, 1∶5000) and S100A4 [15].

### Tumour xenografting

Fresh, surgically excised primary pancreatic adenocarcinoma material was kept in RPMI1640 (Lonza, Switzerland) with 1× Penicillin-Streptavidin (PS) (Lonza) and 0.5 µg/ml Fungizone (Gibco, USA) on ice and transported directly to the animal facility within two hours. The patient material was cut into 2×2 mm pieces and one piece implanted under the skin on each flank of minimum four locally bred female NOD/SCID IL2R **γ** ^0^ (NSG) mice. The general health of the mice was monitored daily and xenograft growth twice a week. The mice were sacrificed when tumours reached 10–15 mm in diameter, and then the xenografts were passaged and/or used to establish *in vitro* cultures. In addition, paraffin-embedded sections were made from early passages.

### Isolation and propagation of human cells *in vitro*

Xenografted tumours were excised when reaching 10 mm and cells were extracted with gentleMACS dissociator (Miltenyi, Germany) according to the manufacturers protocol “*Preparation of single-cell suspensions from implanted mouse tumours, protocol 2.2.2*”. In brief, the xenografts were minced to <5 mm in size and incubated on a tube rotator with 250 U Collagenase I (Worthington, USA) and 0.77 U/ml Dispase (Roche, Switzerland) for 20 minutes at 37°C. Thereafter, samples were run on program “m_impTumor_04” on the dissociator and further rotated for another 20 minutes at 37°C. DNase I (Calbiochem, Germany) was added to a final concentration of 2000 U/ml followed by a last round of the “m_impTumor_04” program. The cell suspension was washed using PEB buffer (0.5% bovine serum albumin (Sigma-Aldrich, USA) and 2 mM EDTA (Lonza)), pelleted by centrifugation at 300 g, resuspended in PBS/2% FCS and filtered through a 40 µM filter to obtain single cells.

To remove mouse cells, magnetic bead depletion was used. The freshly isolated single cell suspension was incubated with mouse anti-H-2Kd antibody (5 µg/10^6^ cells, Becton Dickinson, USA) for 20 minutes at 4°C. Excess antibody was washed away using 2% FCS in PBS (Lonza) by centrifugation at 300 g and the cells were incubated with Dynabeads Pan Mouse IgG (Invitrogen) for 30 minutes at 4°C. Thereafter, the cell suspension was placed onto the magnet for two minutes. The supernatant containing enriched human cells was transferred to a clean tube, washed, pelleted by centrifugation and subsequently resuspended in Pancreatic cell culture medium with 1× pancreatic cell culture supplement ( = “pancreatic medium”) from Millipore, (USA).

The cells were seeded at 20 000 cells/cm^2^ in pancreatic medium and propagated at 37°C, 5% CO_2_. The medium was changed every second or third day and cells were passaged when reaching sub-confluence or every 3^rd^ week, using trypsin/EDTA (Lonza) diluted 1∶5 in PBS. Remaining mouse cells were removed by several washes with PBS after four minutes incubation in trypsin/EDTA during the first passages to avoid them overgrowing the cultures. Pictures of the cultures were taken with Olympus 1×81 with software Cell∧P version 3.3 (Olympus, Germany), or using the Incucyte (Essen Bioscience, UK).

### Flow cytometry analyses and sorting

Subconfluent cell cultures were trypsinized, washed, and resuspended in blocking solution (1 mg/ml human IgG, (Sigma) in PBS), at 4°C for 30 minutes. Cells were pelleted by centrifugation as above and resuspended in blocking buffer with the following monoclonal antibodies, all at concentrations recommended by the manufacturer, TRA-1-85 (clone TRA-1-85, R&D, USA), CD133/1 (clone AC133) and CD133/2 (clone 293C3) both from Miltenyi. H2kD (clone SF1-1.1), CD24 (clone ML5), CD44 (clone G44-26 (C26), CD166 (clone 3A6), CD326 (clone EBA-1), CD184 (clone ID9), SSEA-4 (clone MC813-70), CD15 (clone HI89), all from Becton Dickinson. The cells were incubated at 4°C for 30–45 minutes, washed twice in FACS flow with 2% FCS and resuspended in FACS flow with 2% FCS. Propidium iodide was added to the cells immediately before analysed on a FACS Aria II with FACS Diva software 6.1.3 (Becton Dickinson) or LSR II with FACS Diva software 5.1 (Becton Dickinson).

### Aldehyde dehydrogenase activity and side population assay

Aldehyde dehydrogenase activity was measured using the Aldeflour assay kit (Stem cell technologies, USA) according to manufacturer's protocol. Briefly, 1×10^6^ single cells were put in Aldeflour buffer, Aldeflour reagent was added and half of the cells were immediately transferred to a tube with DEAB inhibitor. Both tubes were incubated at 37°C for 30 minutes. Thereafter, cells were centrifuged, the supernatant discarded and the cells were resolved in cold Aldeflour buffer before beeing analysed by flow as described in the antibody section.

The efflux capacity of cells was measured in a classical side population assay. Briefly, hoechst 33342 (Sigma-Aldrich) was added to 1×10^6^ single cells in 2% FCS to the final concentration of 5 µg/ml and the mixture was incubated at 37°C on a tube rotator at slow speed for 90 minutes. Thereafter, the cells were centrifuged, supernatant discarded and the cells resuspended in ice-cold PBS (Lonza) with 2% FCS and analysed on flow as described above. To provide information of the different efflux pumps active, the following inhibitors was used: Verapamil (50 µM), Reserpine (10 µM) and Fumitremorgin C (10 µM), all from Sigma.

### Generation of colonies in methylcellulose

Cells sorted by flow cytometry or bulk population single cells were plated in methylcellulose (Methocult from Stem Cell Technology, France) mixed with one of the following media: Stem cell medium I: 20 ng/ml EGF (PeproTech, USA), 10 ng/ml bFGF (PeproTech, USA), 1× B27 (Gibco) and 1× PS (Lonza) in 1× Defined Keratinocyte-SFM (Gibco); Stem Cell medium II: 20 ng/ml EGF, 20 ng/ml bFGF, 1× B27, 1× PS and 1× Glutamax (Gibco) in DMEM-F12 (Gibco); Pancreatic medium: Pancreatic culture media with pancreatic cell culture supplement and PS and RPMI medium: 10% FCS, 1× PS and 1× Glutamax in RPMI 1640 (Lonza) according to manufacturers' protocol. In brief, single cell suspensions were added to media with Methocult, mixed and seeded at a density of 1000 cells/well in a non-adhesive 24 well plate (Grenier Bio-One, Germany). Two weeks post plating, the colonies were stained with 150 µl 0.4 mg/ml Thiazolyl Blue Tetrazolium Bromide (MTT) (Sigma-Aldrich) at 37°C for 4 hours or overnight. Uniform colonies of minimum 50 µm were scanned using the GelCount machine and the number and sizes were quantified using GelCount software (both from Oxford Optronix, UK).

### *In vivo* tumourigenicity

Subconfluent cell cultures were trypsinized, washed in RPMI 1640 and filtered to obtain a single cell suspension and thereafter counted in triplet using trypan blue to exclude dead cells. Cells were injected subcutaneously into the flanks of locally bred female NSG mice and tumour size was monitored twice a week.

### Microarray analysis

100 ng total RNA was converted to cDNA, amplified and labelled with Cy-3 using LowInput QuickAmp Labelling Kit (Agilent Technologies, Santa Clara, CA, USA) according to the manufacturer's instructions. The global transcription profiles were evaluated using the SurePrint G3 Human GE 8x60K microarrays (Agilent Technologies, Santa Clara, CA, USA). The mRNA microarray expression profiles from three cell lines with three replicates for two cell lines, four replicates for one cell line, three fresh frozen tumour samples and two corresponding normal tissue samples were background corrected and quantile normalised. Removal of control probes, un-annotated genes and low expressed probes was done by calculating the 95% percentile of the negative control probes on each array. Probes that were at least 10% brighter than the negative controls on at least 50% of the arrays were kept. Further, 6810 differentially expressed mRNAs were removed by carrying out a moderated *t*-test for cell line expression versus tumour samples, since these genes may result from contaminating normal and stromal cells in tumour tissues or proliferation associated mRNAs. The unsupervised hierarchical clustering was carried out using Pearson and Spearman's rank correlation coefficient using 10 948 expressed mRNAs. The data are available through Gene Expression Ominbus (GSE58561).

## Results and Discussion

### Generation of xenografts from pancreatic ductal adenocarcinomas

Due to the limited amounts of surgically removed tissue available and the high percentage of stromal cells in these tumours, we first generated xenografts from the tumour material in NSG mice. In this way, we expanded the tissue and avoided the problem of infiltrating human stromal cells overgrowing the cancer cells. Mouse cells that infiltrated and supported tumour growth could then be removed based on the expression of mouse MHC class I during isolation of cells from the resulting xenograft tumours.

The human samples (Ppa1-9) were obtained from patients with primary pancreatic tumours without known distant metastases, that underwent pancreatoduodenectomy with curative intent. None of the patients had received neoadjuvant treatment. The histological diagnosis of the surgical specimens confirmed that eight of the nine tumours were PDACs, and seven of these were of the expected pancreatobiliary subtype. One tumour, Ppa6, was of the intestinal subtype. The last tumour, Ppa5, was however an intraductal papillary mucinous neoplasia of pancreatobiliary type and was one of the two tumours not able to generate tumours in mice (Table 1 and 2). S100A4- and p53 statuses of the PDAC patient tumours was analysed by immunohistochemistry, while the KRAS status was analysed by real-time PCR. All PDAC tumours had increased levels of p53, although the expression in Ppa1 was weak. All but one PDAC patient sample had KRAS mutations, and the IPMN patient had WT KRAS (Table 2). For the metastasis-associated protein S100A4, we found cells with strong cytoplasmic and/or nuclear expression in patient material Ppa1, Ppa2 and Ppa8 (Table 2). All the original tumours were STR fingerprinted at the OUS genotyping core facility at the Norwegian Radium Hospital (Table S1).

**Table 1:** Characterisation of patient material.

Patient Sample	Gender	Age	Diagnosis^1^	Degree of differentiation^2^	Stage^3^	Resection margin^4^	Disease-free survival (days)	Overall-survival (days)
Ppa1	F	56	PDAC (P)	Poorly	T3	1	325	525
Ppa2	M	63	PDAC (P)	Moderately	T3	1	195	633
Ppa3	M	66	PDAC (P)	Poorly	T3	1	143	481
Ppa4	F	76	PDAC (P)	Moderately	T3	0	286	410
Ppa5	M	72	IPMN (P)	High-grade dysplasia	Tis	0	365#	365#
Ppa6	F	51	PDAC (I)	Moderately	T3	1	184	1228*
Ppa7	F	70	PDAC (P)	Moderately	T3	0	1018*	1018*
Ppa8	F	59	PDAC (P)	Poorly	T3	1	162	634
Ppa9	M	58	PDAC (P)	Moderately	T3	0	985*	985*

1 : PDAC = Pancreatic ductal adenocarcinoma, P = pancreatobiliary subtype, I = intestinal subtype.

IPMN = Intraductal papillary mucinous neoplasia.

2 : The degree of differentiation for each tumour is according to the pTNM Classification of Malignant Tumours [14].

3 : T3 = Tumour extends beyond pancreas, but without involvement of celiac axis or superior mesenteric artery, Tis = Carcinoma *in situ*.

4 : 0 = resection margin free and 1 = resection margin not free.

# : Patient only followed up the first year after operation, due to non-malignant disease.

*: Patient is alive, the number of days given refers to the latest follow-up date.

**Table 2:** Characterisation of tumour mutation status and xenograft tumours.

Patient Sample	KRAS mut	P53^1^	S100A4 cytoplasm^2^	S100A4 nuclear^2^	Xenograft take	Implanted tumours >10 mm^3^	Xenograft growth^4^	Growth *in vitro*
Ppa1	G12V	5 (1)	5 (3)	4 (3)	10/10	13	4–8	yes
Ppa2	G12V	6 (3)	5 (3)	5 (3)	5/24	10	4–9	yes
Ppa3	G12D	6 (3)	5 (3)	5 (3)	10/10	11	8–11	no
Ppa4	G12V	6 (2)	5 (3)	4 (3)	9/10	9	5–10	limited
Ppa5	WT				0/10			
Ppa6	G12R	6 (3)	0 (0)	0 (0)	3/10	28	6–7	yes
Ppa7	WT	5 (2)	4 (1)	0 (0)	7/10	12	8–11	no
Ppa8	G12D	6 (3)	4 (2)	2 (3)	5/8	9	5–8	yes
Ppa9	G12C	6 (3)	5 (1)	0 (0)	0/8			

1 : p53 staining is scored as hot spots where 5 = 30–60% and 6 = 60–100%, and the staining intensity is given in brackets (1 = weak, 2 = medium and 3 = strong).

2 : S100A4 is scored as % positive cells where 1 = 1–4%, 2 = 5–9%, 3 = 10–14%, 4 = 15–49%, 5≥50%, and the staining intensities is given in brackets (1 = weak, 2 = medium and 3 = strong).

3 : Time in weeks required for the implanted patient material to reach 10 mm.

4 :Time in weeks required for the xenograft passage to reach 10 mm for F3 and forward.

Tumours from seven of the nine individual patients gave palpable xenografts within three months after implantation (PpaX1-9 passage F1) (Table 2). Overall, the median time for patient material to establish palpable tumours above 10 mm was 15±6 weeks. There were slight differences between pieces derived from the same tumour (±4 weeks), but these variations were most pronounced between samples from each patient, ranging from 11 to 28 weeks. The variations in time until engraftment could reflect the aggressiveness or proliferation rate of the tumour cells, the fraction of tumourigenic cells within the implanted material and/or the time the tumour cells need to adapt a new environment. Interestingly, the degree of tumour differentiation did not seem to influence the growth rate. However, tumours with poor differentiation had a higher rate of engraftment for the initial operation material, from 90% for poor differentiated tumours to 40% for moderate differentiated tumours (Table 2). This is in agreement with the observation that poorly differentiated adenocarcinomas predicts a poor survival for patients with operable pancreatic cancer [16].

All the initially established xenografts could be serially passaged in mice, and the subsequent passages generally grew faster with more homogenous growth rates between pieces from the same tumour. From the third round of passaging in mouse (F3 and onward), the time before the tumours reached 10 mm stabilized (+/−2.5 weeks) with the average generation time being characteristic for each xenograft line (Table 2). The observed decrease in passaging time during the first passages is in agreement with data from Kim *et al.* [17].

**Figure 1:**
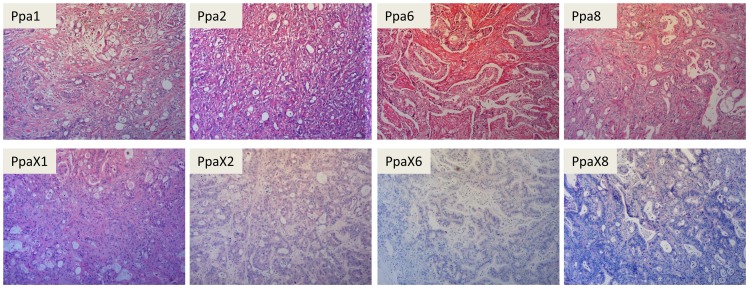
Morphology of the human tumours and the corresponding xenograft tumours. HE-stained sections of original patient material (upper panel) and xenograft tumours at the following passages, PpaX1 p4, PpaX2 p1, PpaX6 p7 and PpaX8 p2, (lower panel) demonstrate the overall conservation of histological features for each tumour pair.

Early passage xenografts were used to make paraffin embedded slides, that later were haematoxylin- and eosin- (HE) stained. These slides were evaluated by a trained pathologist and compared with the original patient sample. In all cases the morphology and differentiation grade were judged to be similar in the xenografts and their corresponding original tumours (Fig. 1).

To further compare the xenografts with the original patient material, we performed immunohistochemistry for selected biomarkers. All examined tumours and xenografts had strong staining for the human-specific antibody against CD326 (EpCAM) and the diagnostic marker cytokeratin 7 (CK7) in the majority of the cells, confirming that all xenografts consisted of human pancreatic cancer cells of epithelial origin (Table 3). Notably, sections from the implanted sites showed that some tumours were able to infiltrate and disrupt the surrounding muscles, which might explain the paralysis observed in some mice carrying PpaX2, PpaX6 or PpaX8 xenografts. Since invasive traits, stemness and tumour aggressiveness may be induced by epithelial-mesenchymal transition (EMT) [18], and expression of EMT markers has been found to be of prognostic value for pancreas cancers [19], expression of EMT-related markers was also investigated. Using a human-specific antibody against the mesenchymal marker vimentin we observed a strong, ubiquitous staining in the stroma of the original tumours in contrast to very few positive stromal cells in the xenografts, demonstrating that very few human stromal cells remain in the xenografts, even in early passages (Table 3). While all original tumours and the other xenografts were negative for vimentin, PpaX2 had a strong, ubiquitous expression of vimentin in the tumour cells, which could suggest that these cells have undergone EMT. All tumours and xenografts had a strong membrane-bound expression for the epithelial marker E-Cadherin in most tumour cells, except the PpA2 original tumour that displayed a weaker staining and in fewer cells (Table 3). Interestingly, the xenografts PpaX1, PpaX2 and PpaX6 all had prevalent, strong nuclear expression of Snail and/or Slug, known inducers of EMT that represses E-Cadherin (Table 3). In contrast, all the original tumours were negative for Snail and/or Slug, and we hypothesize that Snail and/or Slug might be induced by the mouse subcutaneous microenvironment and perhaps orthotopic implantation would be more relevant for *in vivo* EMT studies. A lack of correlation between Snail- and E-Cadherin expression in pancreatic cancers has also previously been reported [20].

**Table 3:** IHC staining of original tumours and xenograft tumours.

Tumours	CK7	EpCAM	E-Cadherin	Snail/Slug^1^	Vimentin
Ppa1	4 (3)	4 (3)	4 (2)	0 (0)	0 (0)
PpaX1	4 (3)	4 (3)	4 (3)	4 (3)	1 (3)
Ppa2	4 (3)	4 (3)	3 (2)	0 (0)	0 (0)
PpaX2	4 (3)	4 (3)	4 (3)	4 (2)	4 (3)
Ppa6	4 (3)	4 (3)	4 (3)	0 (0)	0 (0)
PpaX6	4 (3)	4 (3)	4 (3)	4 (3)	1 (1)
Ppa8	4 (3)	4 (3)	4 (3)	0 (0)	0 (0)
PpaX8	4 (3)	4 (3)	4 (3)	0 (0)	1 (1)

Sections are from the following mouse passage: PpaX1 p4, PpaX2 p1, PpaX6 p7, PpaX8 p2.

% Positive cells are given according to the following system: 1 = 1–10%, 2 = 11–40%, 3 = 41–80%, and 4≥80%, and the staining intensities are given in brackets where (1 = weak, 2 = medium and 3 = strong).

1 : Only cells with nuclear Snail+Slug expression is counted.

### Establishing primary cell lines from the xenografts

When xenograft tumours that had been passaged at least three times reached 10 mm, the mice were sacrificed, tumours excised and single cells extracted using enzymatic and mechanical techniques. Before seeding the isolated cells in regular cell culture flasks, mouse cells were substantially depleted using magnetic beads coated with a mouse MHC class I antibody (H2kD, Fig. S2). Remaining mouse cells were depleted by selective trypsin/EDTA treatment during the establishment of the primary cultures, since human pancreatic cells detach after 15–20 minutes, whereas mouse cells detach fully within 1–4 minutes. Within 3–6 passages the human cells (TRA-1-85 positive) in the cultures was close to 100% and mouse cells (H2kD positive) less than 2%.

The primary cell cultures were sensitive to low seeding densities, in particular in the first passages. Therefore, extracted cells were seeded at high concentration (20 000 cells/cm^2^). To optimize culture viability, maintain the relevant phenotypes and restrain growth of mouse cells, we compared various medium conditions. Initially, we compared the capacity of the cells to grow in different media, like RPMI-1640 with 10% FCS, diverse stem cell media and media specifically designed for pancreatic cells. RPMI-1640 with FCS allowed attachment and growth of the pancreatic cells, however, this medium sustained fibroblastic mouse cells and allowed them to overgrow the culture. All the general stem cell media and most of the pancreatic cell-specific media only allowed a few cells to adhere, and the cell growth was at best very poor. The serum-low Pancreatic Cell Culture Medium ( = “pancreatic medium”) allowed the pancreatic cells to attach and grow and at the same time limited the growth of mouse cells, and it was therefore chosen for further work.

Passaging of the cell lines was also carefully optimized. The cells tolerated neither Accutase nor TrypLE and EDTA alone did not detach the human cells within one hour. Trypsin/EDTA was tolerated when diluted 1∶5 and detached the human cells within 15–20 minutes at 37°C. This was followed by one wash in PBS to remove the remaining trypsin. The resulting primary cell lines grew slowly and were initially passaged every 2–3 weeks. With time, the cells adjusted to growth *in vitro* and in some cases required passaging as often as once a week.

**Figure 2:**
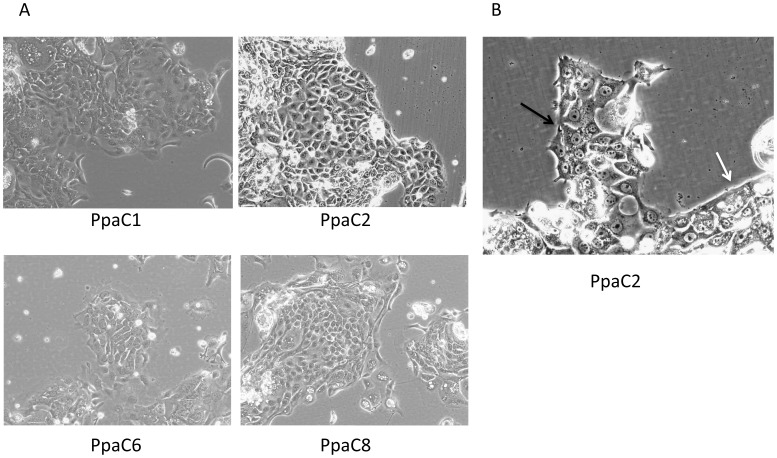
Global mRNA expression pattern. Heat map showing the hierarchical clustering of normal pancreatic tissue, original tumour material and the corresponding cell lines. The data set consists of 10 948 genes after filtering genes commonly regulated in fresh samples or cell lines. The RNA is isolated from the cell lines at the following passages: PpaC1 p16, PpaC6 p11 and PpaC8 p16.

Extracted cells from four of the xenografts (PpaX1, PpaX2, PpaX6 and PpaX8) successfully generated primary cell lines that have been in culture for more than 10 passages *in vitro*, named PpaC1, PpaC2, PpaC6 and PpaC8, respectively. Initially, the cells grew as colonies with typical cobblestone morphology, characteristic for epithelial cells. The colonies were compact with a clear border around the colony (similar to the PpaC2 colony in Fig. 2A). After 3–4 weeks the colony borders weakened and cells grew out of the tight cobblestones in an irregular form and gained a more mesenchymal appearance. At the same time, single cells appeared that generated new colonies. In early passage cultures, the cobblestone morphology prevailed in the cultures, but over time the cultures gradually gained a mesenchymal phenotype with irregular shape growing in a disorganized pattern. This phenomenon was common for PpaC1, PpaC6 and PpaC8 (Fig. 2), however, PpaC2 kept the well-defined cobblestones for a longer time and only partly gained the mesenchymal-like growth appearance. Cultures with cobblestone morphology seemed to preferentially disseminate as cell clusters (Fig. 2B).

To test the ability of the generated cell lines to continuously grow *in vitro*, the PpaC1 culture was propagated for over 30 passages (more than 8 months) without any signs of growth decline; rather it tended to grow faster at higher passages. All four generated cell lines were fingerprinted and the fingerprints matched their corresponding original tumours (Table S1).

### Transcriptome profiles of tumours and corresponding cell lines

**Figure 3:**
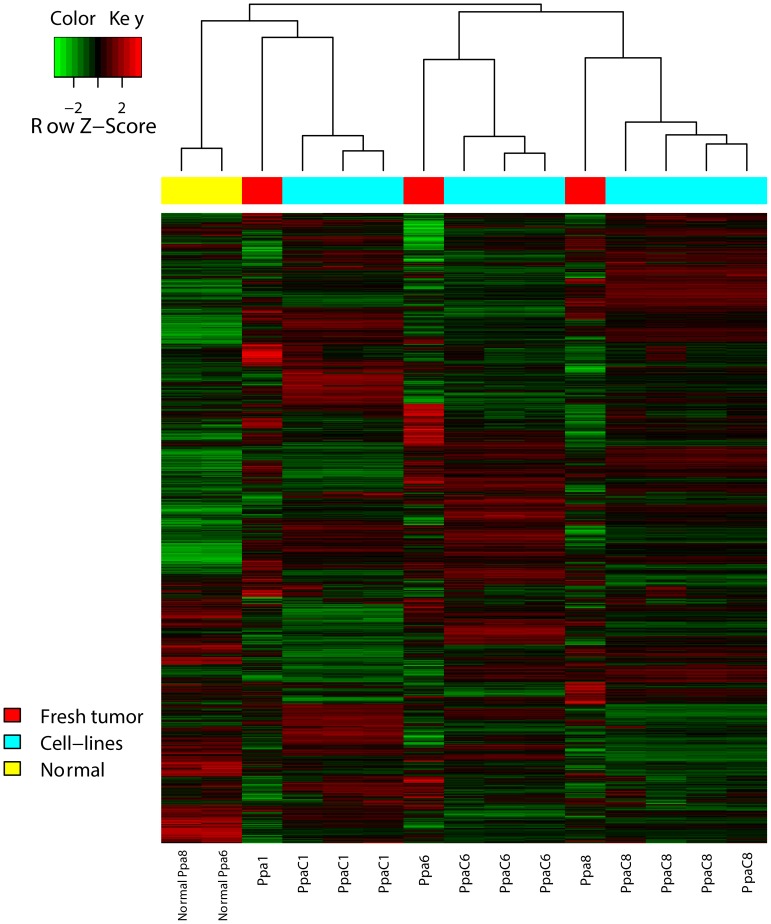
Morphology of the cell lines. A: Phase contrast pictures of the generated cell lines at the following passages: PpaC1 p2, PpaC2 p4, PpaC6 p8 and PpaC8 p10, at 10× magnification. B: Disseminating cell clusters in cultures with cobblestone growth pattern. The regular smooth colony border is indicated with a white arrow and the protruding group of cells with a black arrow. 20× magnification. Picture is from p4.

To investigate whether the cell lines represent the parental tumours we compared the mRNA expression patterns of cells from PpaC1, PpaC6 and PpaC8 with those of the original tumours and also to non-tumourigenic pancreatic tissue from patients Ppa6 and Ppa8. To avoid differences resulting from contaminating normal and stromal cells or proliferation-associated mRNAs overexpressed in culture, we filtered the genes according to Virtanen *et al.* [21]. An unsupervised hierarchical clustering of the filtered mRNA expression patterns using Pearson and Spearman's rank correlation coefficient showed that the cell lines clustered with their corresponding tumour samples while the adjacent normal tissue samples formed a separate cluster (Fig. 3). This demonstrated that although different from the human tumours each cell line shared the overall expression pattern with their corresponding original tumour, supporting the usefulness of the cell lines as models for their unique patient tumours, and also further confirm their origins from the correct patients.

### Colony formation and *in vivo* tumourigenicity

**Figure 4:**
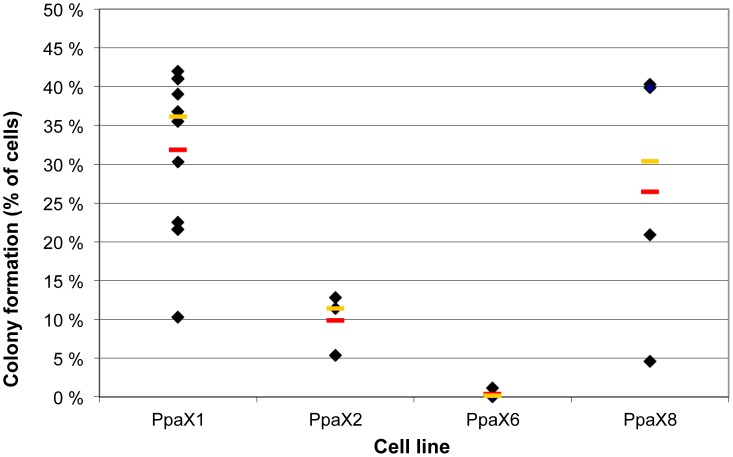
Colony forming capacity of the cell lines. Each diamond represent the fraction of cells able to generate colonies >50 µm in methylcellulose/stem cell medium II in one experiment (n = 3–9). The red line shows the average and the yellow line shows the median colony forming capacity for cells in the following passage span: PpaC1 p1–p31, PpaC2 p5–p13, PpaC6 p2–p7 and PpaC8 p4–p14.

The capacity to form colonies *in vitro* was investigated using anchorage-independent growth in semi-solid methylcellulose. We tested different media also for the colony assays, and despite the fact that Pancreatic medium gave the best growth under regular 2D growth conditions, the stem cell-enriching media gave the highest number of colonies (Table 4). The diameters of the colonies were however similar for all tested conditions. The number of colony-forming cells in the cultures was reproducible but differed markedly between the cell lines, from PpaC1 in which about one of three cells made colonies, to PpaC6 that did not form colonies at all (Fig. 4). The capacity to generate colonies remained stable over passages (Table 4) and it is noteworthy that the two cell lines that gave the highest number of colonies represent cancers with poor differentiation.

**Table 4:** Colony forming capacity of PpaC1.

	*in vitro* passage							
Media	p5	p6	p12	p18	p20	p24	p28	p31
**Stem Cell medium I**	49%	37%	52%	37%	n.d.	42%	35%	n.d.
**Stem Cell medium II**	22%	22%	39%	41%	56%	37%	30%	41%
**RPMI/10%FCS**	18%	7%	24%	n.d.	n.d.	n.d.	n.d.	n.d.
**Pancreatic medium**	10%	n.d.	17%	n.d.	n.d.	n.d.	n.d.	n.d.

The table shows the % of cells able to generate colonies in the indicated semi-solid media in different *in vitro* passages, n.d. = not done.

To confirm that the generated cell lines are tumourigenic, single cell suspensions from the established *in vitro* cultures were injected into the flank of NSG mice without the use of Matrigel. All four cell lines were able to form palpable tumours within three months from as few as 10 000 cells (Table 5). There were no signs of tumours in the 100 or 1000 cell injections for any of the cell lines after six months, which is in agreement with previous reports [6], [7], [22]. Importantly, despite the inability of the PpaC6 cell line to form colonies, it generated tumours in mice as efficiently as the other cell lines. It is however noteworthy that PpaC6 is derived from a tumour of the intestinal subtype while the remaining cell lines were from tumours of the pancreatobiliary subtype.

**Table 5:** *In vivo* tumourigenicity.

Cells injected	PpaC1	PpaC2	PpaC6	PpaC8
**250 000**	4/4	5/5	-	4/4
**100 000**	4/4	5/5	6/6	4/4
**10 000**	4/4	5/5	6/6	2/4
**1 000**	0/4	0/5	0/6	0/4
**100**	0/4	-	0/6	0/4

Shown is the number of tumours growing/the number of injected sites in NSG mice for each cell lines, harvested at the following passages: PpaC1 p6, PpaC2 p12, PpaC6 p6, PpaC8 p13.

### Characterization of stem cells markers

We did not perform clonal selection during generation of the cell lines in order to better conserve the chaotic and multi-mutational cellular hierarchy that exist in human tumours. Accordingly, all the established cell lines had heterogeneous morphologies, which were also reflected in the cell surface expression of known pancreatic and cancer stem cell markers (Table 6). All four cell lines had a high percentage of cells expressing the reported pancreatic cancer stem cell markers CD24, CD44 and CD326 (EpCAM) [6]. The very high number of CD326 positive cells, above 98%, confirms that the cell lines are of epithelial origin, and thus not significantly contaminated by fibroblasts, and also that even the PpaC2 cells had not gone completely through EMT. The expression of CD24 and CD44 varied both between samples and over time, but in general both markers were expressed in more than 50% of the cells in early passages. PpaC1 cells were sorted based on CD24 and CD44 expression and the colony formation capacity as well as the capacity to regenerate all subpopulations upon cultivation was examined. There was no enrichment in the colony forming capacity in the CD24^+^/CD44^+^/CD326^+^ sorted cells (Fig. S3). Furthermore, when we grew the sorted cells for 2–3 passages and reanalysed the CD24 and CD44 expression, each sorted population was able to regenerate all the four subtypes (Fig. S4).

**Table 6:** Cell surface expression of known cancer and stem cell markers.

Marker	PpaC1	PpaC2	PpaC6	PpaC8
**TRA-1-85 +**	96	98	98	98
**H2kd +**	1.7	0.9	1.4	0.1
**CD24**	62	35	97	70
**CD44**	64	64	86	83
**CD133/1**	2.0	45	7	0.6
**CD133/2**	7.7	47	20	5.6
**CD142**	78	87	97	94
**CD166**	57	93	87	69
**CD326/EpCAM**	99	94	100	97
**CD184**	2.1	2.2	1.1	2.4
**SSEA-4**	31	6.0	48	55
**SSEA-1/CD15**	51	48	80	53
**Aldeflour**	3.9	12	13	2.8
**Side population**	4.8	n.d.	n.d	5.8

Shown here is the average % positive cells out of single, live cells, n = at least 3, n.d = not done.

Cells were analysed in the following passage spans: PpaC1 p1–p10, PpaC2 p3–p14, PpaC6 p5–p9 and PpaC8 p4–p20.

To investigate whether certain phenotypes were being selected for or induced during *in vitro* propagation, the expression of some markers was followed during *in vitro* passages for PpaC1. Over time, the percentage of CD44^+^ cells increased and after 13 passages almost all cells had a strong cell surface expression of CD44 (Table S2). We have previously observed this change toward an almost uniform CD44+ cell population also in primary lung cancer cell lines upon passaging [23], but do not know whether this is a selection for growth *in vitro* or an aspect of *in vitro* growth.

Altogether, we could neither find any enrichment of cells with cancer stem cell characteristics based on CD24/CD44/CD326 expression, nor did we observe any hierarchical system for these markers in our cell lines. This is in agreement with our observation that the percentage of cells being able to generate spheroids in semi-solid medium was stable during passages (Table 4), although the percentages of CD44 and CD24 positive cells change over time.

Other markers for cancer stem cells in pancreatic adenocarcinoma have also been suggested, either alone or in combination with one or several of the classical CD24/CD44/CD326 markers. One of these is CD133 that has been investigated in several different cancer types, such as liposarcoma [24], glioblastoma and colorectal carcinoma [25]. In pancreatic adenocarcinoma Hermann *et al.* [7] used CD133 in combination with CD184 to identify migratory cells essential for tumour metastasis.

We investigated the expression of two epitopes of CD133, and both CD133/1 and CD133/2 positive cells could be detected in all the four cell lines with CD133/2 being more abundant. However, there was considerable variation between the cell lines; from 0.6 to 45% for CD133/1, and from 5.6 to 47% for CD133/2 (Table 6), and over time, the frequency decreased during *in vitro* passaging of PpaC1 (Table S2).

In all cell lines there was a distinct population of CD184^+^ cells (Table 6). This is contrary to Jaiswal *et al.* [26] who only found CD184^+^ cells (0.21%) in one of five established cell lines. As for CD133, the percentage of CD184^+^ cells dropped with passaging, suggesting that these markers are not useful *in vitro*.

CD166 is reported to be a cancer stem cell marker in several solid cancers, including pancreatic cancer, although there are conflicting reports regarding its usefulness as a prognostic marker in pancreatic adenocarcinoma. While Khalert *et al.* [27] found that high expression of CD166 in patient samples correlated with poor survival and early tumour relapse, Tachezy *et al.* [28] found no correlation between CD166 expression and patient survival although they did find a higher expression in pancreatic cancer compared to normal tissue. Our cell lines show a high fraction of CD166^+^ cells, in the range of 57–93% (Table 6), which is in accordance with an aggressive phenotype. Furthermore, the CD166^+^ population was stable over time (Table S2).

Another cancer-associated protein is CD142, also known as tissue factor (TF). Although the normal function of CD142 is to initiate the coagulation cascade, it is often highly expressed in cancer tissue [29] as well as in plasma of cancer patients where it can cause thrombosis, a phenomenon known as Trousseau syndrome [30]. When investigated by flow cytometry, the pancreatic cell lines had high fractions (78–97%) of cells with membrane-bound CD142, stable over time (Tables 5 and S2). Important, also the soluble form is reported to be of importance for cancer progression [31].

Both the Side Population (SP) assay, that measures drug efflux capacity, and the Aldeflour assay, that measures the activity of aldehyde dehydrogenase, are widely used to identify or enrich for cells with stem cell-like properties in various cancers, including pancreatic. The PpaC1 and PpaC8 contained SP^+^ subpopulations of similar size (4.8 and 5.8%, respectively) (Table 6), in both cases validated by sensitivity to pre-treatment with the membrane pump inhibitor FTC and also partially with the inhibitors verapamil and reserpine (data not shown). These high percentages of SP^+^ cells are in contrast to the findings of Jaiswal *et al.* [26], who reported less than 1% SP^+^ cells in all their established pancreatic cell lines. This can be due to the long time in culture, but since the side population assay is very sensitive to the metabolic conditions of the cells, this could also be due to small technical differences [32].

The cell lines originating from tumours of moderate differentiation (PpaC2 and PpaC6) had high fractions of Aldeflour^+^ cells (12 and 13% respectively) in contrast to those originating from poorly differentiated tumours (PpaX1 and PpaX8, 2.8 and 3.9% respectively) (Table 6). These numbers are similar to those reported by Kim *et al.* [9] in untreated freshly resected patient material (12.1% and 13.7%), and in the same range as those reported by Rasheed *et al.* [22], [33] for four established pancreatic cell lines (2.4–8.5%), although they reported a lower fraction in a primary pancreatic xenograft (1.7%).

Altogether, we describe a robust procedure for the generation of pancreatic adenocarcinoma cell lines, and a detailed characterisation of four new PDAC xenograft and cell lines. These cell lines are chemotherapy naïve, represent their tumours of origin well, and together with their corresponding xenografts, they represent highly relevant preclinical models for pancreatic cancer.

## Supporting Information

Figure S1 **Schematic figure of the overall working flow.** Implanted tumours were passaged at least two times before cells were extracted to generate *in vitro* cell lines. The cell lines have now been passaged up to 32 times *in vitro*. The cell line passage number used for each cell line in each analysis is indicated in the figure legend of the relevant analysis in the main article.
(PDF)

Figure S2 **Depletion of mouse cells in xenograft single cell suspensions.** Cells were stained with antibodies against the human marker TRA1-85 and the mouse marker H-2Kd before and after depletion of mouse cells in the xenograft single cell suspensions. The percentages of human and mouse cells of live, single cells are indicated in the flow cytometry dot plot diagrams before and after depletion.
(PDF)

Figure S3 **Colony forming capacity in isolated CD24/CD44 cell populations.** CD24/CD44 subpopulations from PPaC1 cells isolated by flow cytometry assisted cell sorting were grown in methylcellulose/stem cell medium II to evaluate their colony forming abilities. The number of colonies (>50 µm)/1000 seeded cells after two weeks are shown.
(PDF)

Figure S4 **Regeneration of CD24/CD44 populations from cultivated CD24/CD44 cell populations.** CD24/CD44 subpopulations from PPaC1 cells isolated by flow cytometry assisted cell sorting were cultured under regular growth conditions for 2–3 passages and reanalysed for the expression of CD24 and CD44. The dot plot to the left shows the original sorting gates, and the four dot plots to the right are the cultivated isolated populations using the color-coding from the original sorted cells. In all dot plots, the Y-axis represent the CD24 expression while the X axis represent the CD44 expression.
(PDF)

Table S1 **STR fingerprinting of human tumours and the generated cell lines.**
(PDF)

Table S2 **Cell surface marker expression during passaging of PpaC1.**
(PDF)
