# Supplementary figures and images for: Generation and Characterisation of Novel Pancreatic Adenocarcinoma Xenograft Models and Corresponding Primary Cell Lines
Source: PLoS One. 2014 Aug 22;9(8):e103873. doi: 10.1371/journal.pone.0103873 (PMC4141735; doi:10.1371/journal.pone.0103873)

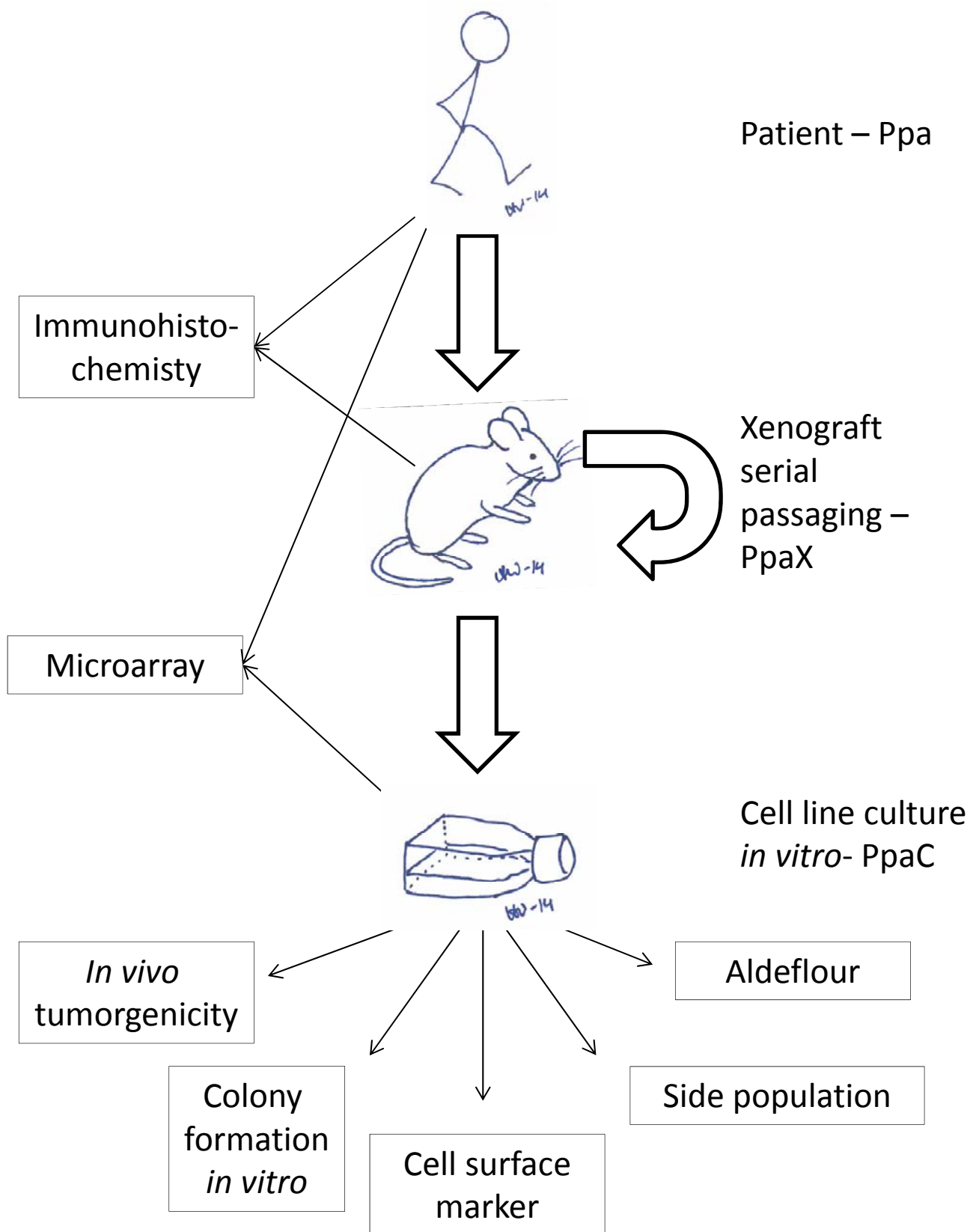

Supplement: Figure S1 — Schematic figure of the overall working flow. Implanted tumours were passaged at least two times before cells were extracted to generate in vitro cell lines. The cell lines have now been passaged up to 32 times in vitro. The cell line passage number used for each cell line in each analysis is indicated in the figure legend of the relevant analysis in the main article. (PDF) [file pone.0103873.s001.pdf]

Fig. S1

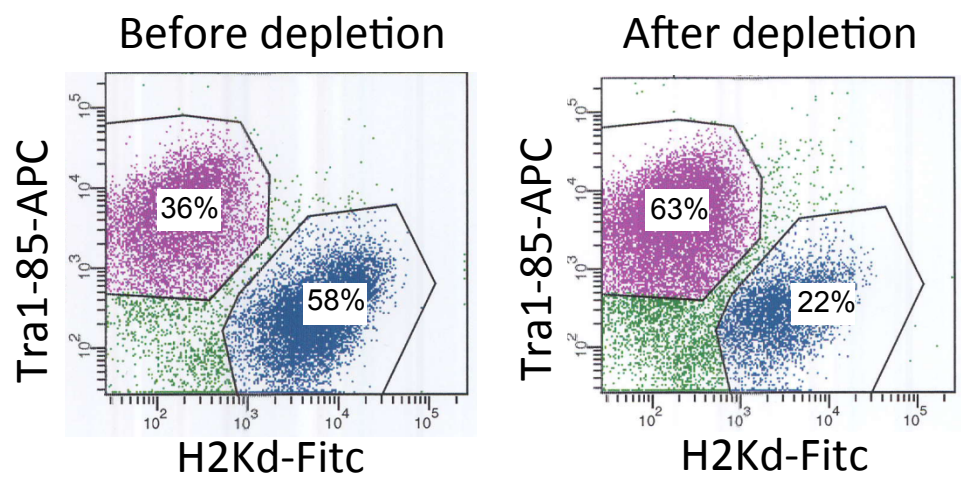

Supplement: Figure S2 — Depletion of mouse cells in xenograft single cell suspensions. Cells were stained with antibodies against the human marker TRA1-85 and the mouse marker H-2Kd before and after depletion of mouse cells in the xenograft single cell suspensions. The percentages of human and mouse cells of live, single cells are indicated in the flow cytometry dot plot diagrams before and after depletion. (PDF) [file pone.0103873.s002.pdf]

Fig S2

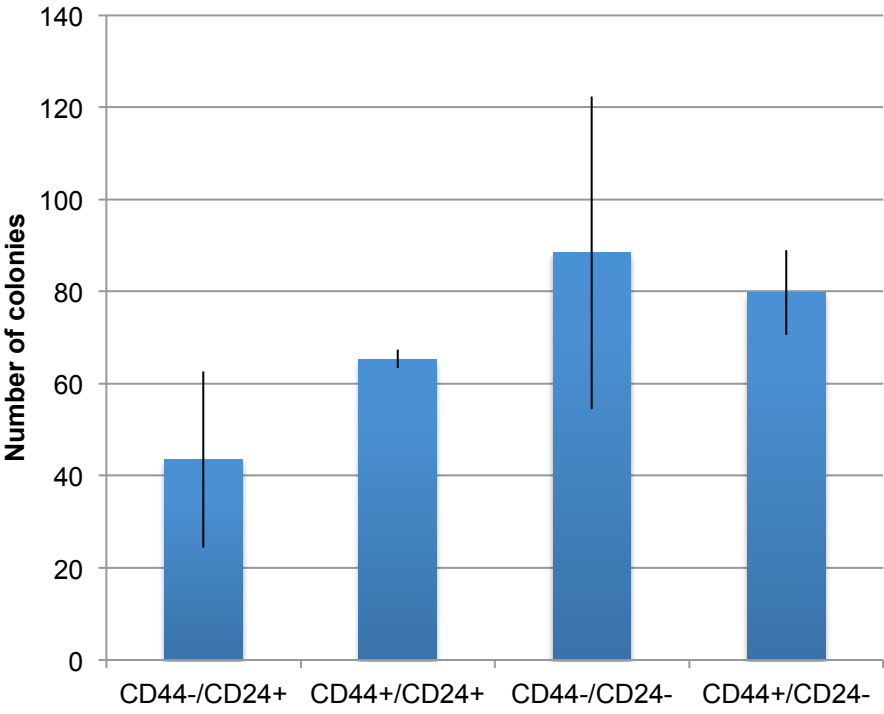

Supplement: Figure S3 — Colony forming capacity in isolated CD24/CD44 cell populations. CD24/CD44 subpopulations from PPaC1 cells isolated by flow cytometry assisted cell sorting were grown in methylcellulose/stem cell medium II to evaluate their colony forming abilities. The number of colonies (>50 µm)/1000 seeded cells after two weeks are shown. (PDF) [file pone.0103873.s003.pdf]

Fig. S3

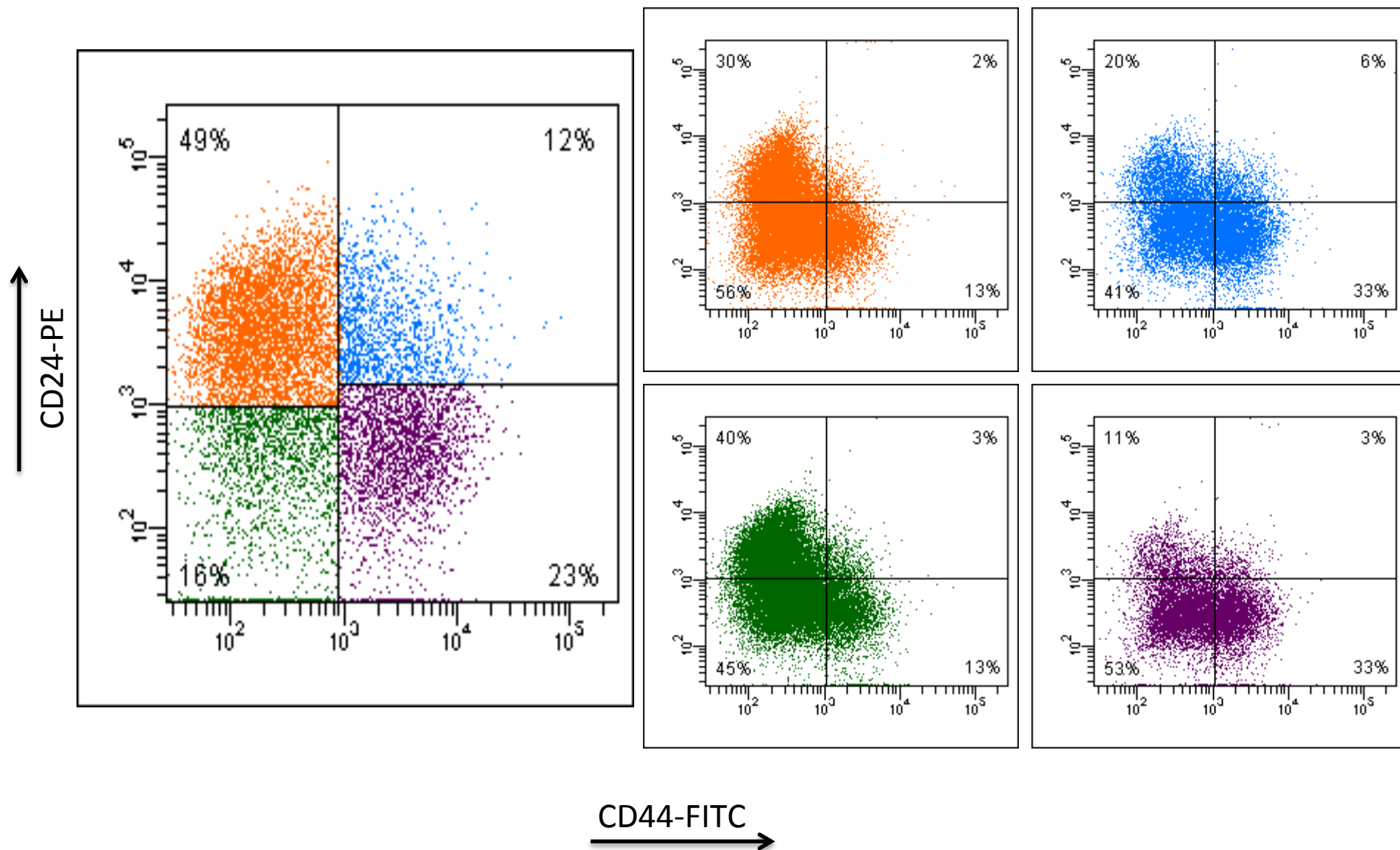

Supplement: Figure S4 — Regeneration of CD24/CD44 populations from cultivated CD24/CD44 cell populations. CD24/CD44 subpopulations from PPaC1 cells isolated by flow cytometry assisted cell sorting were cultured under regular growth conditions for 2–3 passages and reanalysed for the expression of CD24 and CD44. The dot plot to the left shows the original sorting gates, and the four dot plots to the right are the cultivated isolated populations using the color-coding from the original sorted cells. In all dot plots, the Y-axis represent the CD24 expression while the X axis represent the CD44 expression. (PDF) [file pone.0103873.s004.pdf]
