# Supplementary material for: Generation and Characterisation of Novel Pancreatic Adenocarcinoma Xenograft Models and Corresponding Primary Cell Lines
Source: PLoS One. 2014 Aug 22;9(8):e103873. doi: 10.1371/journal.pone.0103873 (PMC4141735; doi:10.1371/journal.pone.0103873)
Supplement: Table S1 — STR fingerprinting of human tumours and the generated cell lines. (PDF) [file pone.0103873.s005.pdf]

Sample	Amelogenin	CSF1PO	D13S317	D16S539	D18S51	D21S11	D3S1359	D5S818	D7S820	D8S1179	FGA	Penta D	Penta E	TH01	TPOX	vWA
Ppa1	x	11	10	6,10,11	14	27.2, 32	16, 17, 21	11	11	12, 14	20, 22	12, 13	7, 12	6	8, 11	16, 18
PpaC1	x	11	10	11	14	28; 32,2	16, 17	11	11	12, 14	20, 22	12,13	7, 12	6	8, 11	18
Ppa2	x, y	10, 13	11, 13	7,11,12	12, 15	27.2, 28.2	14, 17	11, 13	8, 9	11, 13	21, 23	12, 13	10, 20	6, 9.3	8, 11	14, 15
PpaC2	x, y	10, 13	11, 13	12	12	28, 29, 30	14	13	8, 9	11, 13	21,23	12, 13	10, 20	6	8, 11	12, 15
Ppa3	x, y	11, 12	11, 13	13	14, 15	29.2, 30.2	16, 17	11, 12	11	14, 15	18, 21	7, 12	11, 15	9, 9.3	8	15, 16
Ppa4	x	11, 12	8, 9	9, 13	12, 16	27.2, 29.2	16	11	8, 11	14	20, 24	9, 11	11, 14	9, 9.3	8, 11	15, 17
Ppa6	x	11, 12	9, 12	9, 12	14, 16	28.2, 29.2	16	10, 12	8, 10	13	20, 26.2	8, 13	15	4, 7	8, 9	15, 18
PpaC6	x	11, 12	9, 12	9,11,12	14	29	16	10, 12	8, 10	13	20, 26	7, 13	15	7	8, 9	15, 18
Ppa7	x	10, 11	14	6, 11	13, 19	28.2, 29.2	17, 18	11, 13	9, 10	10, 14	20, 21, 22	10, 12	7, 18	9.3	8	16, 18
Ppa8	x	11, 13	9, 12	9, 12	16, 19	28.2	14, 18	11, 13	8, 9	13	21, 23	10, 12	7, 10	4, 7	8	17
PpaC8	x	11, 13	9, 12	9, 12	16	29	14, 18	11, 13	8, 9	13	21, 23	10, 12	7, 10	7	8	17
Ppa9	x, y	11, 12	9, 11	7, 12	13, 15	28.2, 30.2	14, 18	11, 12	10, 13	12, 14	20, 22	9, 12	14, 16	6, 9.3	8, 10	18, 19
